# New-Onset and Exacerbation of Lung Diseases after Short-Term Exposures to Humidifier Disinfectant during Hospitalization

**DOI:** 10.3390/toxics10070371

**Published:** 2022-07-04

**Authors:** Seula Lee, Kyunghee Han, Jeonggyo Yoon, Eun-Kyung Jo, Wonho Yang, Yoon-Hyeong Choi

**Affiliations:** 1 Department of Preventive Medicine, Gachon University College of Medicine, Incheon 21936, Korea; saaa8986@gmail.com (S.L.); wjdry1234@gmail.com (J.Y.); 2 Department of Public Health, Gachon University, Incheon 21936, Korea; vhal1001@naver.com; 3 Department of Community, Environment and Policy, Mel and Enid Zuckerman College of Public Health, University of Arizona, Tucson, AZ 85721-0066, USA; 4 Korean Society of Environmental Health, Seoul 04376, Korea; qhdud7657@gmail.com; 5 Department of Occupational Health, Daegu Catholic University, Gyeongsan 42472, Korea; whyang@cu.ac.kr; 6 Department of Health Science and Technology, GAIHST, Gachon University, Incheon 21999, Korea

**Keywords:** humidifier disinfectants, lung disease, short-term exposure, hospital exposure, PHMG, CMIT/MIT, PGH

## Abstract

(1) Background: Humidifier disinfectant (HD) is a biocidal chemical to keep the water tank inside a humidifier clean. Thousands of Koreans have experienced HD-related lung injuries. Of them, 6.9% were exposed to HD in hospitals. (2) Methods: This study investigated changes of diseases in patients (or caregivers) who experienced HD exposures during hospitalization and also investigated characteristics of hospital exposure using data from all HD-related lung injury enrollment in Korea. (3) Results: Of a total of 162 subjects, 139 subjects were hospitalized for non-lung diseases, and 23 people were hospitalized for lung diseases at the time of hospitalization. During hospital exposure, 99 (71.2%) of those hospitalized with non-lung disease experienced a new-onset of lung disease, and 15 (65.2%) of those hospitalized with lung diseases experienced exacerbation of their existing lung diseases. When we compared their exposure characteristics, those exposed in hospitals (vs. non-hospital, mostly home) were exposed for shorter periods, at closer distances, at higher HD indoor concentrations, constantly all day, and directly in the facial direction. (4) Conclusion: In conclusion, HD exposures in hospital with a high intensity even for a short term were associated with new-onset or exacerbation of lung diseases. Our findings suggest that acute exposures to HD can cause lung diseases.

## 1. Introduction

Humidifier disinfectant (HD) is a biocidal product that has been widely used in South Korea to prevent the growth of microorganisms, germs, and molds in the water tank of a humidifier. HD was first developed in 1994. It is estimated that approximately 9.5 million HD products (25 or more kinds of HD products) have been sold [1,2,3]. Major chemical ingredients known to be contained in HD products are polyhexamethylene guanidine (PHMG, CAS#: 89697-78-9), chloromethylisothiazolinone (CMIT, CAS#: 26172-55-5), methylisothiazolinone (MIT, CAS#: 2682-20-4), oligo-(2-(2-ethoxy)-ethoxyethyl) guanidine chloride (PGH, CAS#: 374572-91-5), and sodium dichloroisocyanurate (NaDCC, CAS: 2893-78-9) although major chemicals may differ depending on the product [4,5,6]. The sale of HD products was officially banned in 2011 when their lung toxicity was identified [7]. Several experimental studies have reported that HD chemicals could induce apoptosis, inflammation, and fibronectin expression in alveolar epithelial cells and trigger a wound-healing response, leading to epithelial stiffness and thickening, eventually leading to lung diseases [8,9,10]. A review of epidemiologic studies has reported a causal association between HD exposure and the risk of HD-related lung injury (HDLI) [11]. Furthermore, a study of lung injury cases suggested a higher risk of lung injury in people associated with HD use than those with insecticide uses, indoor molds, and only humidifier uses (without HD) at home [12], and a previous study suggested that HD exposure was associated with lung diseases, including idiopathic interstitial pneumonia [13].

To date, a total of 7525 subjects are enrolled in the Korea Ministry of Environment for compensation due to HD-related adverse health effects. Among them, 4177 subjects were confirmed to be HDLI cases, including 1017 deaths [14].

In addition to its use in households, HD was used in healthcare facilities such as hospitals, local clinics, nursing homes, and postnatal care centers. Indeed, a previous study reported that 6.9% subjects with HDLI-suspected enrollment might have been exposed to HD during hospitalization [15]. That study suggested a possibility that people exposed to HD during hospitalization likely had a humidifier placed right next to their bed (i.e., high concentrations at a close distance) and were exposed to HD all day without breaking time (i.e., almost 24 h) because of their inactive behaviors, such as sedentary, reclining, and lying posture, for several days to months. Given such exposure characteristics in hospitals and the vulnerability of patients, patients exposed to HD in hospitals might newly develop lung diseases, such as pneumonia or pulmonary fibrosis, during hospitalization and have an exacerbation of their lung diseases existing at the time of admission.

Therefore, the aim of this study was to investigate in the short term the changes of diseases status (particularly exacerbation of existing lung disease and new-onset of lung disease) in patients and patients caregivers who experienced HD exposures during hospitalization, which might be a certain environment with high-level of HD exposures at a close range and for a short period (i.e., days-to-months) and to also compare the exposure characteristics between hospital exposures and non-hospital exposures using exposure-assessment data of HDLI-suspected subjects enrolled in the Korea Ministry of Environment.

## 2. Materials and Methods

### 2.1. Study Population

This study used data from the HD-exposure assessment among the subjects who had been exposed to HD and were suspected of having HDLI that were enrolled in the Korea Ministry of Environment. The HD-exposure assessment is an ongoing, nationwide investigation conducted by the Korea Centers for Disease Control and Prevention (KCDC) supervised by the Korea Ministry of Health and Welfare from July 2013 to March 2014 (Cycle I) and subsequently conducted by the Korea Environmental Industry and Technology Institute (KEITI), supervised by the Korea Ministry of Environment from July 2014 to present (Cycle II to IV) [14]. Exposure assessment of HD was designed to assess HD exposures in each enrolled subject and to provide evidence to determine a link between HD and its health effects. Data were collected through extensive interview using structured HD-specific questionnaires regarding demographic characteristics, HD exposures characteristics (including place of HD use, exposure period, and exposure proximity), and HD-related diseases [15,16,17,18]. This exposure assessment was approved and reviewed by the Institutional Review Board of Daegu Catholic University (IRB number: CUIRB-2016-0114). All subjects provided written informed consent before participation.

Of all enrolled subjects (*n* = 6059 participants) in all four cycles of the HD exposure assessment, there were 360 subjects who had experienced HD exposures at hospitals (142 participants with exposure at hospital only and 218 participants with exposure at hospital and non-hospital) and 5699 participants who had exposure at non-hospital only.

**Figure 1:**
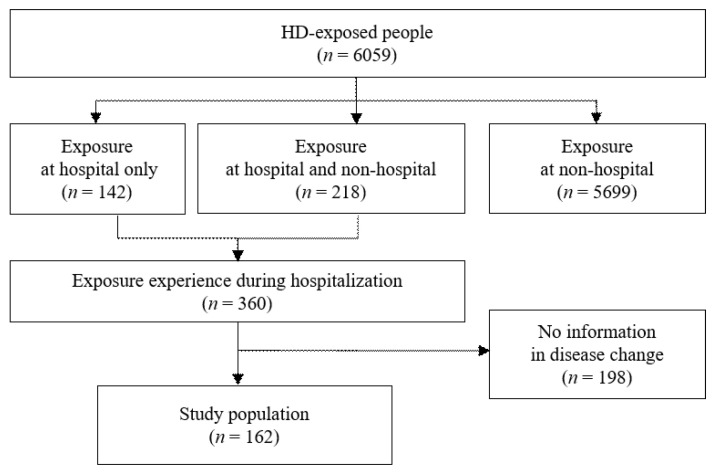
Flow chart showing the selection of study population.

**Figure 2:**
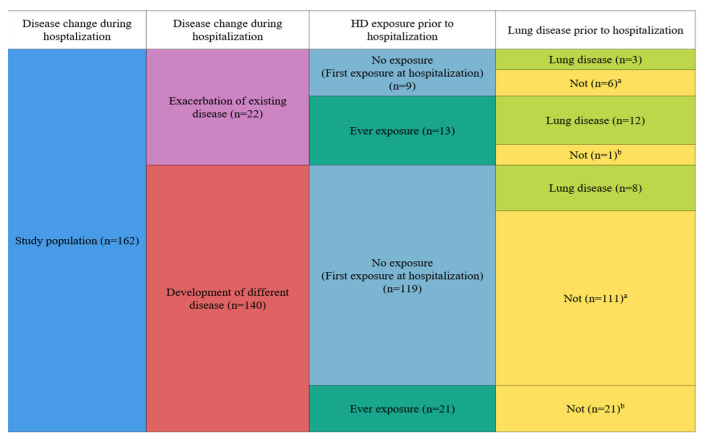
Classification of participants. Subjects number of each group: 128 for no exposure (first exposure at hospitalization) group and 34 for ever-exposure group; 23 for lung disease group and 139 for no lung disease group. ^a,b^ Subjects shown in Supplementary Tables S2 and S3.

After excluding subjects without available information of disease status changes during hospitalization, the current study included a total of 162 participants (Figure 1). Detailed classifications of participants by disease status changes during hospitalization and HD exposure prior to hospitalization are shown in Figure 2.

### 2.2. Disease Change during Hospitalization

All subjects provided information about their diseases before and after hospitalization and self-reported their physician diagnosis. We evaluated disease status change and disease classification change. Disease status change was classified into cases with exacerbation of existing diseases and cases with development of different disease (Figure 2). Exacerbation of existing disease was defined when diseases subtypes were not changed during hospitalization, but the symptoms worsened (progressed), or the disease became severe. Development of different diseases was considered when additional comorbidity was newly diagnosed after hospitalization.

Disease classification change was the change between the disease classification at the time of hospitalization and the disease classification after exposure to HD at hospitals. Disease classification was categorized depending on organ system: lung, not lung (respiratory disease and others), and none (caregiver). Lung disease included subtypes of pneumonia, pulmonary fibrosis, lung function decline, lung transplantation, and lung cancer. Respiratory disease included subtypes of apnea, asthma, and otorhinolaryngologic disease. Others mean all other diseases, including neurogenic diseases, bone fractures, proctological disorders, and brain diseases. Detailed subtypes of each disease classification are shown in Supplemental Table S1.

### 2.3. Humidifier Disinfectant Exposure

In order to estimate individual exposure to HD, we considered the following six exposures criteria: exposure proximity, exposure direction, daily exposure time, exposure duration, cumulative exposure time, and airborne exposure intensity. Exposure proximity was defined as the distance from the humidifier to face. It was categorized into <1 m or ≥1 m. Exposure direction was defined as the spray direction of HD. It was categorized as toward the face or other sides. Daily exposure time (hour) was defined as the total time of exposure to HD. Exposure duration was defined as the total period of exposure to HD. Cumulative exposure time (hour) was calculated by multiplying years of use (year), annual use (months/year), monthly use (weeks/month), daily use (days/week), and hourly use (hours/day). Indoor air concentration (μg/m^3^) was calculated by multiplying the used HD product concentration by daily average usage amount and dividing by the volume of the space where it was used according to a previously reported method [19,20].

Furthermore, we computed distance-adjusted indoor air concentration (μg/m^3^) because exposure concentration at a close range might differ from the exposure concentration at a long range. European Chemical Agency has reported a method of distance-adjusted indoor air concentration for sprayed products such as biocides [21], which is similar to the airborne exposure to HD. Because the total amount of the sprayed product enters immediately and homogenously the volume of air surrounding the subject (i.e., 2 m^3^), the volume of the room can be reduced to represent the personal volume rather than the volume of a whole room. In the current study, therefore, when the participant was exposed within 0.5 m, the individual exposure was considered to have been in the volume of 2 m^3^ regardless of the actual room volume. Additionally, we also evaluated it with a similar but more conservative method suggested by Weerdesteijn et al. (i.e., 5 m^3^) [22].

In addition, exposure experience prior to hospitalization was classified as ever or not. “Ever” exposure was defined when the subject had experienced any exposures to HD prior to hospitalization, and “non” was defined when subject had never experienced HD exposure prior to hospitalization (Figure 2).

### 2.4. Characteristics of Participants

In the current study, we considered the following characteristics of participants: survival status, age, sex, cigarette smoking, and education level. Survival status of enrolled subject was defined as either survivor or not (referred to as survivor and death). Age was categorized as ≤6, 7–19, 20–64, and ≥65 years old. Participant’s age for survivor was defined as when participant experienced HD-related health effects. Participant’s age for death was defined as when participant died after HD exposure. Education level was categorized as ≤elementary school, middle school, high school, and ≥college graduate. Cigarette smoking was categorized as never, former, or current smoker.

### 2.5. Statistical Analysis

SAS version 9.4 (SAS Institute, Cary, NC, USA) was used for all statistical analyses. *p*-value was two sided. Statistical significance was considered at *p* < 0.05. In descriptive analyses, data are presented as mean ± standard deviation (SD) for continuous variables and the number of participants and proportion (%) for categorical variables. For testing differences of participants’ characteristics, Student’s *t*-test was used for continuous variables, and χ^2^ test (or Fisher’s exact test) was used for categorical variables.

## 3. Results

Table 1 presents characteristics of all participants. Among a total of 162 subjects, there were 96 (59.3%) males and 52 (32.1%) current survivors. The mean age ± SD was 53.40 ± 25.74 years. For HD exposure characteristics, 132 (85.7%) subjects were exposed to HD at distance less than 1 m, and 136 (87.2%) subjects were exposed to HD toward the face. Mean ± SD values of exposure duration, cumulative exposure time, and indoor air concentration were 29.52 ± 32.99 months, 14,642.88 ± 19,871.96 h, and 547.99 ± 662.20 μg/m^3^, respectively. There were 128 (79.0%) subjects without HD exposure prior to hospitalization (i.e., first exposure to HD during hospitalization). During hospitalization, 22 (13.6%) subjects showed exacerbation of existing diseases, and 140 (86.4%) subjects had new development of different diseases.

**Figure 3:**
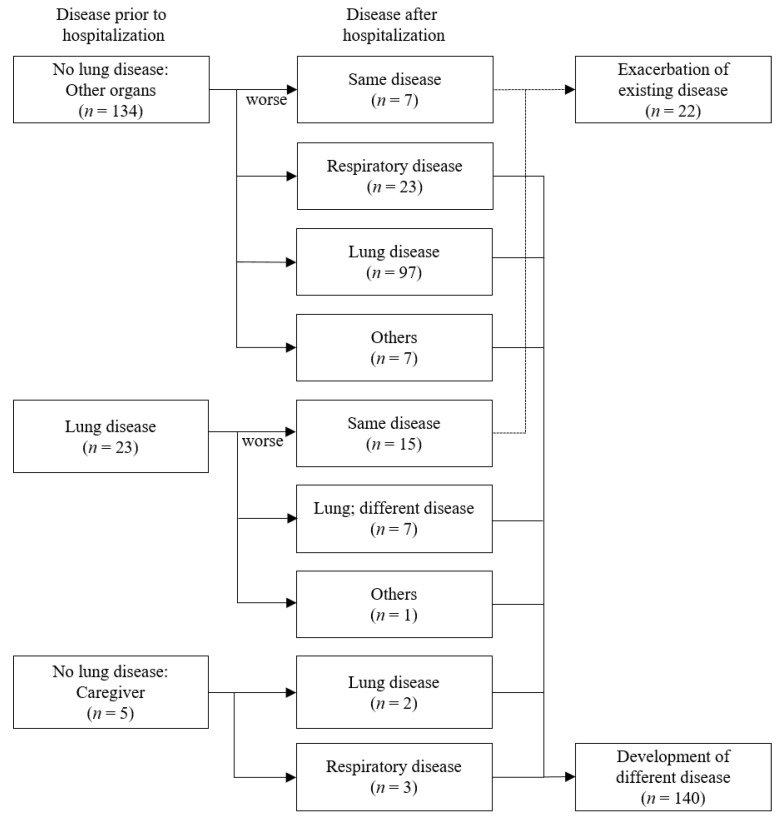
Changes in disease status before and after hospitalization.

Figure 3 presents changes in disease status prior to and after hospitalization. Of a total of 162 subjects, 139 subjects (patients with no lung disease and caregivers) had no lung disease (i.e., other organ diseases and none) prior to hospitalization, and 23 subjects had lung diseases prior to hospitalization. Of these 139 subjects without lung diseases prior to hospitalization, 99 (71.2%) subjects had new-onset of lung diseases during hospitalization, and 26 (18.7%) subjects had new development of respiratory disease after hospitalization. Of those 23 subjects with lung diseases prior to hospitalization, 15 (65.2%) subjects had an exacerbation of existing lung diseases during hospitalization.

When we classified changes in disease status into two groups (exacerbation of existing diseases and development of different diseases), 22 (13.6%) subjects experienced an exacerbation of existing disease, and 140 subjects (86.4%) experienced new development of different disease during hospitalization (Table 2). Compared to subjects with exacerbation of existing disease (*n* = 22), those with development of new different diseases (*n* = 140) were likely to be older and had non-exposure prior to hospitalization (first exposure during hospitalization). Additionally, when we compared subjects with lung disease development (*n* = 99) and those without lung disease development (*n* = 40), there were significant differences in sex and age but not in HD exposure characteristics (Supplemental Table S2).

In sensitivity analysis, we investigated the subpopulation (*n* = 117) after excluding subjects who had experienced HD exposure prior to hospitalization and observed similar results (Supplemental Figure S1 and Table S3).

Table 3 presents participants’ characteristics of subjects with exposure at hospital only (*n* = 142) and those with exposure at non-hospital (*n* = 5699) to compare HD exposure characteristics between hospital exposure and non-hospital exposure. Subjects with exposure at both hospital and other places (*n* = 218) were not included in this analysis because it was impossible to indicate which of these two places had exposure characteristics in the current data. Compared to subjects with non-hospital exposure, subjects with hospital exposure only had significantly higher age and lower education level. They were likely to be now dead and never or former smokers. When we compared HD exposure characteristics, subjects with hospital exposure only (vs. non-hospital exposure) were likely to be exposed to HD at a short distance (<1 m), toward the face, for the whole day (24 h), and for shorter duration. Moreover, indoor air concentration (μg/m^3^), estimated as a homogeneous concentration in a whole room, was significantly lower for subjects with hospital exposure only (vs. non-hospital), while distance-adjusted indoor air concentrations (μg/m^3^), estimated as higher concentration at a close distance, was significantly higher in subjects with hospital exposure (vs. non-hospital). There was no significant difference in cumulative exposure time.

## 4. Discussion

The current study investigated changes of diseases status in patients and patients’ caregivers who had experienced HD exposure during hospitalization using exposure-assessment data of HDLI-suspected cases enrolled in the Korea Ministry of Environment. We found that many patients hospitalized due to lung diseases had their lung diseases worsened during the hospital stay and that many patients hospitalized due to other organ diseases and/or patients’ caregivers developed new lung diseases during the hospital stay, in which hospital ward rooms had a certain environment of high-level HD exposure at a close range. The fact that the majority of the study subjects had either development or exacerbation of lung diseases during hospital exposure can be explained by the environmental characteristics of hospital exposure, which are different from those of HD exposure at home. Indeed, our study found that people exposed in hospitals (vs. non-hospital, mostly home) were exposed for shorter periods of time, at closer distances, at higher HD ambient concentrations, constantly all day, and directly in the facial direction. First, constant exposure throughout the day suggests a possibility that lungs may continue to deteriorate without time to recover. In fact, a previous study indicated that the risk of HDLI is strongly associated with recurrent and intense exposures of acute HD without sufficient time to recover, and its risk might be possibly more than the risk with long-term exposure at chronic to HD [18]. Second, the fact that patients in hospital beds were exposed directly in the facial direction at closer distances might lead to more severe effects on their lungs even though they did not have lung diseases when they were initially hospitalized. Thus, our results suggest that even a short period of exposure can cause enough lung diseases (i.e., lung injury, pulmonary fibrosis, pneumonia, lung cancer, pulmonary function decline, lung transplantation, pneumothorax, and tuberculosis) if exposed to HD at a high intensity.

In particular, this study found that subjects exposed in hospital were exposed to HD at more concentrated indoor levels compared to those exposed in a non-hospital setting using a exposure-assessment method with distance correction. The South Korean government requires that at least 70% of total beds in general hospitals be in multi-person rooms (four or more people, with large volume) [23]. Indeed, more than 90% of total beds are multi-bed rooms [24]. Because HD chemicals emitted from one corner of a hospital room could not spread homogeneously in a large space, a traditional exposure-assessment method (that is, a whole volume is assumed as homogeneous concentration) may not reflect a subject-specific exposure. Thus, we used a method suggested by the European Chemical Agency (that is, exposure to the sprayed product should be assumed as exposure in the personal volume, i.e., 2 m^3^ regardless of the actual room volume). We observed that patients who were exposed to HD in hospitals had significantly higher HD concentration in inhaled air. Therefore, if HD exposure is highly concentrated, it would be risky even for a short period of time.

Although major chemicals contained in HD may differ depending on the products, they are known to PHMG, CMIT/MIT, PGH, or NaDCC [4,5,6]. There are multiple sources of epidemiological evidence showing that exposure to airborne HD chemicals via inhalation might lead to the development of lung diseases [12,24,25]. Those chemicals may share an underlying mechanism capable of inducing inflammation, leading to an elevated fibrotic response. One in vivo study of PGH showed that exposure through intratracheal instillation in mice can increase cytokine and fibronectin levels [10]. In vivo studies of PHMG also showed that exposure through inhalation and intratracheal instillation in rats can induce the release of pro-inflammatory cytokines and cause fibronectin mRNA expression and histopathological changes in lung tissues [26,27]. In vitro studies of PHMG and CMIT/MIT suggested that their exposures can induce the release of proinflammatory cytokines in macrophages and alveolar epithelial cells in mice, respectively [8,28]. Experimental studies on PGH and PHMG using human alveolar epithelial cells also suggested that their exposure can increase levels of fibrotic responses [9]. Therefore, HD chemicals may induce inflammation and fibronectin mRNA expression and cause epithelial cells to stiffen and thicken, eventually leading to pulmonary fibrosis.

The important strength of this study was that we used data of all suspected cases of HDLI in Korea and selected all available subjects who were exposed to HD in hospitals. Second, the high rate of lung disease reported in hospital exposure was explained by the difference in exposure characteristics between hospitals and non-hospitals in this study unlike other studies, which approached differences in vulnerability by demographic characteristics of subjects themselves.

Despite these strengths, this study also has several limitations. Most limitations were derived from the nature of the data, in which all participants only included the subjects who were exposed to HD (regardless of exposure place) as well as suspected of having HDLI. First, our findings might not be able to refer to a general population, including HD-exposed people and un-exposed people. Second, we could not compare the risks of health outcomes between exposed people and non-exposed people. Third, there was a possibility of potential recall bias and measurement error to estimate the exposure characteristics including duration and proximity because exposure assessment of HD was based on participants’ memory for HD use in the past although a well-structured exposure assessment was performed. Fourth, the change in disease status was defined not as physician diagnosis but self-reported, and we cannot rule out a potential misclassification of disease status. Because their health effects might be in the progress as being determined as caused by HD exposures or not, some of them might be diagnosed as no HDLI. Fifth, there is a possibility that it may not be clear whether the change in disease was due to the HD exposure or the natural progression of a disease. Finally, since those exposed in hospitals were observed to be older than those in non-hospitals, we could not rule out the possibility that they were more vulnerable and therefore had greater health effects even with the same level of HD exposure.

## 5. Conclusions

In conclusion, this study suggests that HD exposures in hospitals with high intensity even for a short period of time might be associated with new-onset or exacerbation of lung diseases. Our findings contribute to epidemiological evidence that acute exposures to HD could lead to lung diseases.

## Figures and Tables

**Table 1:** Characteristics of participants (*n* = 162).

Characteristics	Value
Age (years) ^a^	53.40 ± 25.74
≤6	17 (8.6)
7–19	8 (7.4)
20–64	62 (37.0)
≥65	70 (46.9)
Sex	
Male	96 (59.3)
Female	66 (40.7)
Survival status	
Survivor	52 (32.1)
Death	110 (67.9)
Cigarette smoking ^a^	
Never smoker	101 (62.7)
Former smoker	56 (34.8)
Current smoker	4 (2.5)
Education level ^a^	
≤Elementary school	59 (38.6)
Middle school	25 (16.3)
High school	42 (27.5)
≥College	27 (17.6)
Exposure prior to hospitalization	
Ever	34 (21.0)
Non (first exposure during hospitalization)	128 (79.0)
Disease after hospitalization	
Lung disease development	121 (74.7)
No development	41 (25.3)
Disease status change	
Exacerbation of existing disease	22 (13.6)
Development of different disease	140 (86.4)
HD exposure characteristics ^a^	
Exposure proximity	
<1 m	132 (85.7)
≥1 m	22 (14.3)
Exposure direction	
Toward the face	136 (87.2)
Toward the other sides	20 (12.8)
Daily exposure time	
<24 h	51 (35.9)
24 h (whole day)	91 (64.1)
Exposure duration (month)	29.52 ± 32.99
Cumulative exposure time (h)	14,642.88 ± 19,871.96
Indoor air concentration ^b^ (μg/m^3^)	547.99 ± 662.20

Data are presented as mean ± SD for continuous variables and sample size (percentage) for categorical variables. ^a^ Subsample of participants with available information for each characteristic. ^b^ Indoor air concentration was calculated with the following formula: [HD amount used at every injection × chemical concentration contained in the HD product]/room volume.

**Table 2:** Characteristics of participants by disease status change during hospitalization.

Characteristics	Exacerbation of Existing Disease (*n* = 22)	Development of Different Disease (*n* = 140)	*p*- Value ^a^
Age (years) ^b^	34.76 ± 26.30	56.28 ± 24.51	<0.001
≤6	6 (28.6)	11 (8.1)	<0.001
7–19	2 (9.5)	6 (4.4)	
20–64	11 (52.4)	51 (37.5)	
≥65	2 (9.5)	68 (50.0)	
Sex			0.986
Male	13 (59.1)	83 (59.3)	
Female	9 (40.9)	57 (40.7)	
Survival status			0.976
Survivor	7 (31.8)	45 (32.1)	
Death	15 (68.2)	95 (67.9)	
Cigarette smoking ^b^			0.292
Never smoker	16 (72.7)	85 (61.2)	
Former smoker	5 (22.7)	51 (36.7)	
Current smoker	1 (4.5)	3 (2.2)	
Education level ^b^			0.612
≤Elementary school	5 (26.3)	54 (40.3)	
Middle school	3 (15.8)	22 (16.4)	
High school	7 (36.8)	35 (26.1)	
≥College	4 (21.1)	23 (17.2)	
Exposure prior to hospitalization			<0.001
Ever	13 (59.1)	21 (15.0)	
Non (first exposure during hospitalization)	9 (40.9)	119 (85.0)	
Disease after hospitalization			0.450
Lung disease development	15 (68.2)	106 (75.7)	
Not development	7 (31.8)	34 (24.3)	
HD exposure characteristics ^b^			
Exposure proximity			0.523
<1 m	18 (81.8)	114 (86.4)	
≥1 m	4 (18.2)	18 (13.6)	
Exposure direction			1.000
Toward the face	19 (86.4)	117 (87.3)	
Toward the other sides	3 (13.6)	17 (12.7)	
Daily exposure time			0.665
<24 h	11 (50.0)	40 (33.3)	
24 h (whole day)	11 (50.0)	80 (66.7)	
Exposure duration (month)	30.35 ± 34.66	24.68 ± 20.69	0.297
Cumulative exposure time (h)	11,772.0 ± 11,148.70	15,227.7 ± 21,205.10	0.275
Indoor air concentration ^c^ (μg/m^3^)	524.7 ± 690.80	637.7 ± 553.10	0.588

Data are presented as mean ± SD for continuous variables and sample size (percentage) for categorical variables. ^a^ *p*-value based on chi-square test or Fisher’s exact test. ^b^ Subsample of participants with available information for each characteristic. ^c^ Indoor air concentration was calculated with the following formula: [HD amount used at every injection × chemical concentration contained in the HD product]/room volume.

**Table 3:** Characteristics of participants by exposure place.

Characteristics	Hospital Exposure only (*n* = 142)	Non-Hospital Exposure only (*n* = 5699)	*p*-Value ^a^
Age (years) ^b^	60.31 ± 21.62	32.29 ± 25.29	<0.001
≤6	11 (7.8)	1980 (34.7)	<0.001
7–19	5 (3.5)	302 (5.3)	
20–64	49 (34.5)	2724 (47.8)	
≥65	77 (54.2)	693 (12.2)	
Sex			0.229
Male	82 (59.1)	2916 (51.2)	
Female	60 (40.9)	2754 (48.3)	
Unknown	0 (0.0)	29 (0.5)	
Survival status			<0.001
Survivor	50 (35.2)	4600 (80.7)	
Death	92 (64.8)	1099 (19.3)	
Cigarette smoking ^b^			<0.001
Never smoker	12 (9.2)	216 (4.3)	
Former smoker	47 (35.9)	1000 (20.1)	
Current smoker	72 (55.0)	3760 (75.6)	
Education level ^b^			0.001
≤Elementary school	52 (44.8)	1533 (34.0)	
Middle school	17 (14.7)	484 (10.7)	
High school	31 (26.7)	1055 (23.4)	
≥College	16 (13.8)	1436 (31.9)	
HD exposure characteristics ^b^			<0.001
Exposure proximity			
<1 m	108 (87.1)	3751 (66.7)	
≥1 m	16 (12.9)	1871 (33.3)	<0.001
Exposure direction			
Toward the face	110 (85.9)	3790 (68.4)	
Toward the other sides	18 (14.1)	1748 (31.6)	
Daily exposure time			<0.001
<24 h	21 (18.8)	4597 (82.8)	
24 h (whole day)	91 (81.3)	953 (17.2)	
Exposure duration (month)	14.01 ± 26.75	29.25 ± 29.39	<0.001
Cumulative exposure time (h)	7601.40 ± 16,658.30	10,193.20 ± 13,487.80	0.116
Indoor air concentration ^c^ (μg/m^3^)	314.40 ± 249.50	956.00 ± 4017.30	<0.001
Adjusted indoor air concentration ^d^ (μg/m^3^)	8490.7 ± 7116.2	3933.4 ± 12,115.8	0.012
Adjusted indoor air concentration ^e^ (μg/m^3^)	3438 ± 2798.1	1977.4 ± 5791.7	0.037

Data are presented as mean ± SD for continuous variables and sample size (percentage) for categorical variables. ^a^ *p*-value based on chi-square test or Fisher’s exact test. ^b^ Subsample of participants with available information for each characteristic. ^c^ Indoor air concentration was calculated with the following formula: [HD amount used at every injection × chemical concentration contained in the HD product]/room volume. ^d^ Proximity-adjusted indoor air concentration (ECHA, 2015). ^e^ Proximity-adjusted indoor air concentration [22].

## Data Availability

The data that support the findings of this study are available from the corresponding author upon reasonable request.

## Supplementary Materials

The following are available online at https://www.mdpi.com/article/10.3390/toxics10070371/s1, Table S1: Classification of diseases; Table S2: Characteristics of participants with lung disease development or without lung disease development during hospitalization in subpopulation excluding subjects with lung disease prior to hospitalization (*n* = 139 ^a^); Table S3: Characteristics of participants with lung disease development or without lung disease development during hospitalization in subpopulation excluding subjects with lung disease and ever exposure prior to hospitalization (*n* = 117 ^a^); Figure S1: Changes in disease status before and after hospitalization excluding subjects with ever exposure prior to hospitalization.
