# Supplementary material for: New-Onset and Exacerbation of Lung Diseases after Short-Term Exposures to Humidifier Disinfectant during Hospitalization
Source: Toxics. 2022 Jul 4;10(7):371. doi: 10.3390/toxics10070371 (PMC9318961; doi:10.3390/toxics10070371)
Supplement: Supplementary file 1 [file toxics-10-00371-s001.zip › toxics-1722420-supplementary.pdf]

Supplemental Table S1. Disease classification.

Disease	
Lung disease	Lung injury, pneumonia, pulmonary fibrosis, lung function decline, lung cancer, tuberculosis, pneumothorax
Respiratory disease	Apnea, asthma, tonsillitis, chronic bronchitis
Others	Neurogenic disease, bone fracture, proctological disorder, brain disease, ascites, kidney disease, enteritis, urinary tract infection, cardiovascular disease, leukemia, anorexia nervosa, other cancer (stomach cancer, laryngeal cancer, tonsil cancer, colon cancer, thyroid cancer, breast cancer, esophageal cancer), gastrointestinal disorder, peritonitis, gynecological diseases, mental illness, muscular skeletal disease, liver cirrhosis, sepsis, multiple organ failure

Supplemental Table S2. Participants' characteristics by lung disease development during hospitalization in subpopulation excluding subjects with lung disease prior to hospitalization (n = 139[a]).

Characteristics	Lung disease development (n=99)	Not development (n=40)	*P*-value[b]
Age (years)[c]	58.75 ±24.08	47.16 ±24.42	0.001
≤6	7 (7.2)	4 (10.8)	0.174
7-19	5 (5.2)	1 (2.7)	
20-64	33 (34.0)	19 (51.4)	
≥65	52 (53.6)	13 (35.1)	
Sex			0.011
Male	63 (63.6)	16 (40.0)	
Female	36 (36.4)	24 (60.0)	
Survival status			0.015
Survivor	28 (28.3)	20 (50.0)	
Death	71 (71.7)	20 (50.0)	
Cigarette smoking[c]			0.124
Never smoker	58 (59.2)	30 (75.0)	
Former smoker	38 (38.8)	9 (22.5)	
Current smoker	2 (2.0)	1 (2.5)	
Education level			0.065
≤Elementary school	40 (43.0)	12 (30.8)	
Middle school	16 (17.2)	8 (20.5)	
High school	27 (29.0)	8 (20.5)	
≥College	10 (10.8)	11 (28.2)	
Exposure prior to hospitalization			0.087
Ever	19 (19.2)	3 (7.5)	
Non (first exposure during hospitalization)	80 (80.8)	37 (92.5)	
HD exposure characteristics[c]			
Exposure proximity			0.082
<1 m	83 (88.3)	29 (76.3)	
≥1 m	11 (11.7)	9 (23.7)	
Exposure direction			0.535
Toward the face	82 (85.4)	34 (89.5)	
Toward the other sides	14 (14.6)	4 (10.5)	
Daily exposure time			0.185
<24 hr	33 (38.4)	9 (25.7)	
24 hr (whole day)	53 (61.6)	26 (74.3)	
Exposure duration (month)	23.49±22.11	41.70±45.52	0.025
Cumulative exposure time (hr)	11728.10±12020.80	19869.60±27658.20	0.118
Indoor air concentration[d] (μg/m³)	536.60±746.40	519.90±371.50	0.920

Data in tables are mean ± SD for continuous variables and sample size (percentage) for categorical variables.
[a]Subjects a+b in Figure 2.
[b]*P*-value based on Chi-square test or Fisher's exact test.
[c]Subsample of participants with available information for each characteristic.
[d]Airborne exposure intensity was calculated by [HD amount used at every injection × chemical concentration contained in the HD product]/room volume.

Supplemental Table S3. Participants' characteristics by lung disease development during hospitalization in subpopulation excluding subjects with lung disease and ever exposure prior to hospitalization (n = 117[a]).

Characteristics	Lung disease development (n=80)	Not development (n=37)	*P*-value[b]
Age (years)	60.56 ±23.28	48.41 ±24.00	0.013
≤6	4 (5.1)	3 (8.8)	0.181
7-19	4 (5.1)	1 (2.9)	
20-64	25 (32.1)	17 (50.0)	
≥65	45 (57.7)	13 (38.2)	
Sex			0.050
Male	48 (60.0)	15 (40.5)	
Female	32 (40.0)	22 (59.5)	
Survival status			0.008
Survivor	21 (26.3)	19 (51.4)	
Death	59 (73.8)	18 (48.6)	
Cigarette smoking[c]			0.386
Never smoker	48 (60.8)	27 (73.0)	
Former smoker	29 (36.7)	9 (24.3)	
Current smoker	2 (2.5)	1 (2.7)	
Education level			0.091
≤Elementary school	35 (46.7)	11 (30.6)	
Middle school	13 (17.3)	7 (19.4)	
High school	18 (24.0)	7 (19.4)	
≥College	9 (12.0)	11 (30.6)	
HD exposure characteristics[c]			
Exposure proximity			0.026
<1 m	69 (92.0)	27 (77.1)	
≥1 m	6 (8.0)	8 (22.9)	
Exposure direction			0.751
Toward the face	68 (88.3)	32 (91.4)	
Toward the other sides	9 (11.7)	3 (8.6)	
Daily exposure time			0.694
<24 hr	19 (27.9)	8 (24.2)	
24 hr (whole day)	49 (72.1)	25 (75.8)	
Exposure duration (month)	23.16±22.27	42.21±47.24	0.031
Cumulative exposure time (hr)	12923.00±13010.20	19581.50±28571.50	0.233
Indoor air concentration[d] (μg/m^3)	572.40±854.20	572.10±353.10	0.999

Data in tables are mean ± SD for continuous variables and sample size (percentage) for categorical variables.
[a]Subjects a in Figure 2.
[b]*P*-value based on Chi-square test or Fisher's exact test.
[c]Subsample of participants with available information for each characteristic.
[d]Airborne exposure intensity was calculated by [HD amount used at every injection × chemical concentration contained in the HD product]/room volume.

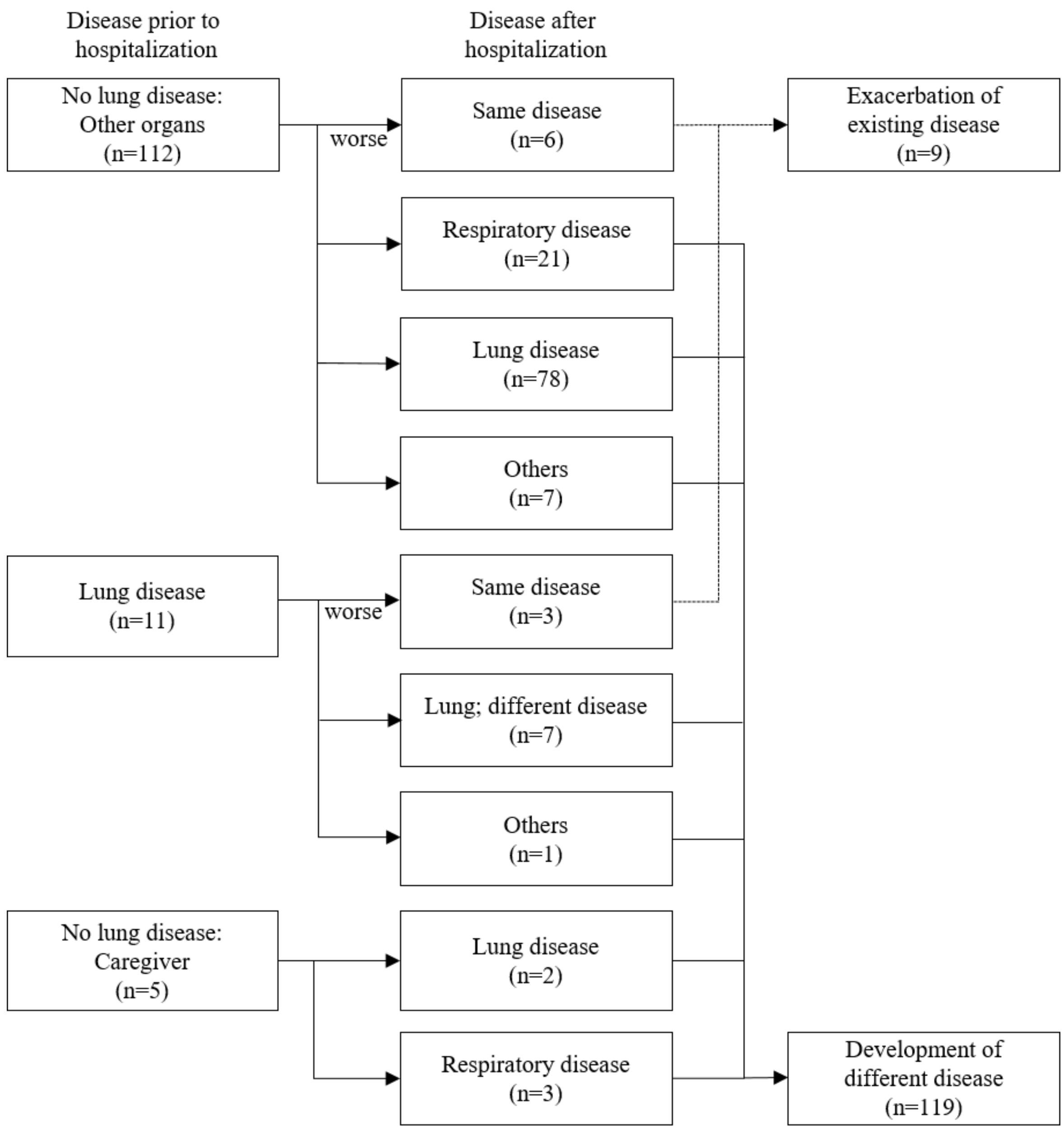

Supplemental Figure S1. Change in disease status prior to and after hospitalization excluding subjects with ever exposure prior to hospitalization.
